# Small intestinal bacterial overgrowth is associated with irritable bowel syndrome and is independent of proton pump inhibitor usage

**DOI:** 10.1186/s12876-016-0484-6

**Published:** 2016-07-11

**Authors:** Evangelos J. Giamarellos-Bourboulis, Emmanouel Pyleris, Charalambos Barbatzas, Aikaterini Pistiki, Mark Pimentel

**Affiliations:** 4th Department of Internal Medicine, National and Kapodistrian University of Athens, Medical School, Athens, Greece; Department of Gastroenterology, Sismanogleion Athens General Hospital, Marousi, Greece; GI Motility Program, Cedars-Sinai Medical Center, Los Angeles, CA USA

**Keywords:** Small intestinal overgrowth, Proton pump inhibitors, Irritable bowel syndrome

## Abstract

**Background:**

Current knowledge suggests that small intestinal overgrowth participates in the pathogenesis of irritable bowel syndrome. It is questionable if this association is modulated by intake of proton pump inhibitors (PPIs).

**Methods:**

In a prospective study, quantitative cultures of duodenal aspirates were performed for aerobic species in 897 consecutive patients undergoing upper GI tract endoscopy. SIBO was defined as equal to or more than 10^3^ cfu/ml. The effect of PPI intake on the relationship between SIBO and IBS was the primary endpoint.

**Results:**

Analysis among patients without any history of PPI intake (*n* = 713) showed that odds ratio (OR) for IBS in the event of SIBO was 5.63 (3.73–8.51, *p* < 0.0001); this was 4.16 (1.91–9.06) when analysis was done among patients with history of PPI intake (*n* = 184, p: 0.498 between patients without and with PPI intake). Multiple logistic regression analysis found that factors independently associated with SIBO were age above or equal to 60 years (OR: 2.36), body mass index more than or equal to 22 kg/m^2^ (OR: 0.60), presence of IBS (OR: 6.29), type 2 diabetes mellitus (OR: 1.59) and gastritis (OR: 0.47).

**Conclusions:**

The association between IBS and SIBO was completely independent from PPI intake. Although gastritis was protective against SIBO, results show that PPI intake cannot prime SIBO.

## Background

Several studies published the last few years support an association between small intestinal bacterial overgrowth (SIBO) and irritable bowel syndrome (IBS) [1–4]. SIBO represents the overgrowth of bacterial species that usually predominate in the large bowel in the proximal small intestine. As a result of SIBO, fermentation of dietary carbohydrates by these bacteria leads to overproduction of gas and to the generation of symptoms of IBS. Most of existing studies linking SIBO and IBS use for the diagnosis of SIBO lactulose or glucose breath tests [1–4]. However the gold standard of diagnosis is the culture of the content of the proximal part of the small intestine. In a recent survey of our group, 320 consecutive patients undergoing upper GI tract endoscopy were studied. Fluid was collected from the third part of the duodenum and quantitatively cultured. Using a cut-off of 10^3^ cfu/ml of colonic type bacteria, the frequency of SIBO was 19.4 %. SIBO was significantly linked with odds ratio 5.64 with the presence of IBS (*p* < 0.0001) [5].

To dates, a number of papers suggest that the linkage between SIBO and IBS could be an epiphenomenon of the chronic intake of proton pump inhibitors (PPIs) leading to changes of the intestinal pH and promoting the colonization by large intestinal flora [6, 7]. In a recent meta-analysis, an association was found between intake of PPIs and development of SIBO only for studies comparing incidence of SIBO before and post-treatment with PPIs within the same population but not when comparing with an independent control population [8]. Available studies do not comment on the type of PPI intake, the dose regimen and the existence of an association between IBS and PPI intake [6–8]. As a consequence, great heterogeneity is generated if indeed an association between SIBO and PPI intake exists. Our cohort of 320 patients using forward step-wise logistic regression analysis showed that the linkage between SIBO and IBS was independent from the intake of PPIs [5]. Over the years, this prospective cohort has been expanded to 904 patients. We aim, using this cohort of 904 patients including the previously published 320 patients, to provide a definitive answer on the association between SIBO and PPI based on the gold standard for SIBO and to determine the association between IBS and PPIs as well.

## Methods

### Study design

This prospective study took place during the period September 2009 to March 2013. Patients who were subject to upper GI tract endoscopy in the Department of Gastroenterology of Sismanogleion General Hospital of Athens were eligible for the study. Every patient was allowed to be enrolled once after written informed consent. The study protocol was approved by the Ethics Committee of the Sismanogleion General Hospital of Athens.

Inclusion criteria were: a) age ≥ 18 years; b) written informed consent; and c) clinical indication for upper GI tract endoscopy. Exclusion criteria were: a) infection by the human immunodeficiency virus; b) chronic infection by the hepatitis B and hepatitis C viruses; c) Child Pugh liver cirrhosis stages 2 and 3; d) active GI tract bleeding; e) gastroesophageal reflux disease (GERD); f) systemic sclerosis; g) any antibiotic intake the last one month prior to endoscopy; and h) inflammatory bowel disease.

Fluid from the third portion of the duodenum was collected during upper GI tract endoscopy. In case of patients with little fluid in the duodenum, the endoscope was approached close to the intestinal wall to allow the aspiration of the biggest possible amount of fluid. Never water was flushed in the duodenal lumen before completion of fluid aspiration. The samples were immediately transported and quantitatively cultured using serial dilutions in sterile NaCl 0.9 % under aerobic conditions. An aliquot of 0.1 ml was plated onto MacConckey agar (Becton Dickinson, Conckeysville Md) and incubated for 18 h at 35^0^C. Bacterial growth was determined after multiplying the number of isolated bacteria with the respective dilution factor. Identification of bacteria was done as described previously [5].

The following information was recorded for each patient: age, gender, height, weight, reason for endoscopy, endoscopic findings, other diseases and intake of any medication. Specifically, full information regarding the type and the duration of PPI intake was registered. A patient was considered to have history of PPI intake if he/she had been administered one PPI for at least the last one month on a daily basis by his/her case-history. Adherence to PPI treatment was based on case-history and daily dose and start and stop dates were confirmed by the information provided in the prescription system for every single patient.

Based on their history, patients were classified as suffering from IBS if they met the following criteria: recurrent abdominal pain or discomfort at least 3 days per month in the previous 3 months associated with 2 or more of the following [9]: i) Improvement with defecation; ii) onset associated with a change in frequency of stool; and iii) onset associated with a change in form (appearance) of stool.

Patients with IBS were further sub-classified based on their symptoms into three bowel habit subtypes ie diarrhea-predominant (IBS-D); constipation-predominant (IBS-C); and alternating bowel habit (IBS-A) according to the Rome criteria [10].

The primary study endpoint was the relationship between SIBO and IBS in light of the history of PPI intake. The secondary study endpoint was the impact of PPI intake on the characteristics of SIBO and IBS. Since a total of 320 patients were needed in a previous study of our group to disclose a significant relationship between SIBO and IBS [5] and making the hypothesis that 30 % of totally enrolled patients will have history of PPI intake, to analyse the association between SIBO and IBS separately among patients without PPI intake and among patients with PPI intake, it was assumed that a total of 900 patients should be enrolled.

### Statistical analysis

In order to explore the primary study endpoint, SIBO was defined in three different ways using three different cut-offs of concentrations of colonic type bacteria in the duodenal aspirate ie >10^3^, >10^4^ and >10^5^ cfu/ml. Comparisons of qualitative variables between patients with SIBO and those without SIBO was done by the Chi-square test. Odds ratios (ORs) and 95 % confidence intervals (CIs) were calculated by the Mantel-Haenzel’s statistics. ORs were compared by the Tarone’s and Breslow-Day’s tests. Comparisons of quantitative variables were done by the Student’s “*t*-test”. In order to define a cut-off value for a quantitative variable that can discriminate SIBO from non-SIBO with specificity greater than 90 %, receiver operator curve (ROC) analysis was done. Then forward step-wise logistic regression analysis was performed taking into consideration all variables differing between SIBO and non-SIBO patients at a *p* value lower than 0.100 and the intake of PPIs as well. Regarding the secondary endpoints, patients were divided into those with history and into those without history of PPI intake. Comparisons between these two subgroups comprised the counts of proximal intestine colonizers where the type of PPI, the duration of PPI intake and the clinical subtype of IBS were taken into consideration. 2-sided *P* values less than 0.05 were considered significant.

## Results

### Primary study endpoint

**Fig. 1:**
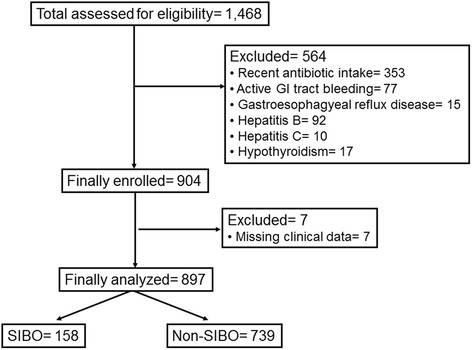
Study flow chart. Abbreviations: GI; gastrointestinal; SIBO: syndrome of intestinal bacterial overgrowth

Fully available data were missing in seven patients and analysis was done in a total of 897 patients (Fig. 1). From the total analysed patients, 184 (20.5 %) had a history of recent PPI intake. The overall frequency of SIBO was 17.6 % when the ≥10^3^ cfu/ml diagnostic cut-off was used; it was 15.6 % when the ≥10^4^ cfu/ml diagnostic cut-off was used; and it was 10.6 % when the ≥10^5^ cfu/ml diagnostic cut-off was used. Comparative characteristics between patients with SIBO and patients without SIBO are shown in Table 1. Overall, endoscopic findings were negative for 410 patients. No differences were found between the two groups of patients regarding history of PPI intake. However, patients with SIBO were older, they had a greater frequency of IBS, of type 2 diabetes mellitus (T2DM) and of anemia and a lower frequency of endoscopic presence of gastritis.

**Table 1:** Demographic characteristic of enrolled patients in the prospective cohort

	No SIBO (*n* = 739)	SIBO (*n* = 158)	*p*
Age (years, mean ± SD)	63.3 ± 17.0	69.7 ± 17.4	<0.0001
Age ≥60 years (n, %)	475 (64.3)	129 (81.6)	<0.0001
BMI (kg/m^2^, mean ± SD)	26.5 ± 5.3	25.6 ± 5.4	0.071
BMI ≥22 kg/m^2^ (n, %)	637 (86.5)	128 (81.0)	0.064
Presence of IBS (n, %)	159 (21.5 %)	93 (58.9 %)	<0.0001
Type of IBS (n, %)			
Predominant-diarrhea	44 (6.0)	33 (20.9)	<0.0001
Predominant-constipation	28 (3.5)	8 (5.1)	0.382
Mixed type	87 (11.8)	52 (32.9)	<0.0001
Co-morbidities (n, %)			
Type 2 diabetes mellitus	171 (23.1)	53 (33.5)	0.008
Chronic heart failure	192 (26.0)	49 (31.0)	0.200
Chronic obstructive pulmonary disease	74 (10.0)	16 (10.02)	1.000
Chronic renal disease	20 (2.7)	8 (5.1)	0.131
Solid tumor malignancy	67 (9.1)	11 (7.0)	0.441
History of drug intake (n, %)			
PPIs	149 (20.2 %)	35 (22.2 %)	0.588
Non-steroidal anti-inflammatory drugs	30 (4.1 %)	11 (7.0)	0.139
Low-dose aspirin	139 (18.8)	34 (21.5)	0.438
Acenocoumarone	63 (8.5 %)	15 (9.5)	0.644
H2-blockers	15 (2.0)	2 (1.3)	0.751
Antacids	23 (3.1)	8 (5.1)	0.230
Clinical reason for gastroscopy (n, %)			
Dyspepsia	422 (57.1)	94 (59.5)	0.596
Anemia	327 (44.2)	83 (52.5)	0.065
Unknown fever	21 (2.8)	7 (4.4)	0.312
Endoscopic findings (n, %)			
Gastritis	388 (52.5)	58 (36.7)	<0.0001
Duodenal ulcer	46 (6.2)	12 (7.6)	0.481
Gastric ulcer	9 (1.2)	1 (0.6)	1.000

An analysis between presence of SIBO and presence of IBS was conducted where all diagnostic cut-offs of isolation of bacteria from the duodenal aspirate were considered ie ≥10^3^ cfu/ml, ≥10^4^ cfu/ml and ≥10^5^ cfu/ml. Whatever the threshold was, the OR for IBS between patients with SIBO was greater compared to patients without SIBO. In all cases, the OR for acquisition for IBS in the setting of SIBO was not greater for patients with a positive history of PPI intake than patients without PPI intake (Table 2).

**Table 2:** Linkage between SIBO and PPI intake

Cut-off of SIBO	History of PPI intake		SIBO (-)	SIBO (+)	*p*	OR (95 % CIs)	p between ORs^a^
	No	Non-IBS	478 (81.0 %)	53 (43.1 %)			0.498
≥10^3^ cfu/ml		IBS	112 (19.0 %)	70 (56.9 %)	<0.0001	5.63 (3.73–8.51)	0.499
	Yes	Non-IBS	102 (68.5 %)	12 (34.3 %)			
		IBS	47 (31.5 %)	23 (65.7 %)	<0.0001	4.16 (1.91–9.06)	
	No	Non-IBS	482 (79.9 %)	49 (44.5 %)			0.712
≥10^4^ cfu/ml		IBS	121 (20.1 %)	61 (55.5 %)	<0.0001	4.95 (3.24–7.59)	0.712
	Yes	Non-IBS	104 (67.5 %)	10 (33.3 %)			
		IBS	50 (32.5 %)	20 (66.7 %)	0.001	4.16 (1.81–9.54)	
	No	Non-IBS	497 (77.8 %)	34 (45.9 %)			0.892
≥10^5^ cfu/ml		IBS	142 (22.2 %)	40 (54.1 %)	<0.0001	4.12 (2.51–6.75)	0.892
	Yes	Non-IBS	107 (65.6 %)	7 (33.3 %)			
		IBS	56 (34.3 %)	14 (66.7 %)	0.007	3.82 (1.46–10.01)	

^a^by the Tarone’s and Breslow-Day tests respectively

Analysis (Table 3) revealed that age ≥60 years, presence of IBS and type 2 diabetes mellitus were positively linked with SIBO. BMI ≥22 kg/m^2^ and endoscopic gastritis were protective from SIBO. Intake of PPI was not related with SIBO. To exclude the possibility that gastritis was a confounding factor in analysis, the frequency of SIBO was analyzed in 451 patients without endoscopic findings of gastritis; 358 had no history of PPI intake and 23 had a history of PPI intake; frequency of SIBO was 21.5 and 24.7 % respectively (p: 0.296).

**Table 3:** Logistic regression analysis of factors independently related with SIBO

	OR	95 % CIs	*p*
Age ≥60 years	2.36	1.45–3.84	0.001
BMI ≥22 kg/m^2^	0.60	0.36–0.99	0.049
Presence of IBS	6.28	4.26–9.25	<0.0001
Type 2 diabetes mellitus	1.59	1.04–2.45	0.032
Intake of PPIs	0.76	0.45–1.42	0.765
Anemia	1.24	0.84–1.84	0.271
Endoscopic gastritis	0.47	0.32–0.69	<0.0001

Abbreviations: *CI* confidence interval, *OR* odds ratio

### Secondary endpoints

**Fig. 2:**
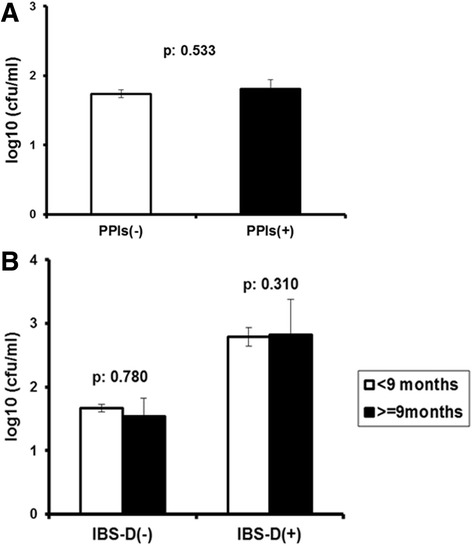
Lack of association between bacterial growth in duodenal aspirates and history of PPI intake **a** Bacterial growth in patients without history of PPI intake [PPIs(-)] and patients with history of PPI intake [PPIs(+)]; **b** Bacterial growth in patients with IBS predominant diarrhea [IBS-D(+)] and the rest of patients [IBS-D(-)] in relation to the time of PPI intake. *P* values show differences of the indicated comparisons

Analysed patients were divided into those without history of recent PPI intake (*n* = 713) and into those with history of recent PPI intake (*n* = 184). The absolute counts of bacteria in the duodenal aspirates did not differ between groups (Fig. 2a).

Preliminary analysis (data now shown) revealed that the use of PPIs for ≥9 months prior to endoscopy can be related with the presence of IBS at specificity greater than 90 %. As shown in Fig. 2b, no differences in bacterial counts of the duodenal aspirates could be found between patients with less than or ≥9 month-history of PPI intake assigned either to the IBS-D subgroup or to the IBS non-diarrhea subgroup. The same applied for the type of bacteria isolated from the duodenal aspirates. No differences were found between patients without and with history of PPI intake (Table 4). Moreover, the positive association between SIBO and IBS remained constant irrespective of the type of PPI intake (Table 5).

**Table 4:** Impact of PPI intake on the type of bacteria of the duodenal aspirates in relation to the presence of IBS or not

	Type of bacteria	No PPI intake (%)	PPI intake (%)	*p*
SIBO without IBS (*n* = 65)	*Escherichia coli*	11 (20.7)	2 (16.7)	
	*Klebsiella pneumoniae*	11 (20.7)	2 (16.7)	
	*Enterobacter cloacae*	7 (13.2)	0 (0)	
	*Staphylococcus aureus*	6 (11.3)	2 (16.7)	
	*Enterococcus faecalis*	3 (5.7)	1 (8.3)	
	*Pseudomonas aeruginosa*	3 (5.7)	0 (0)	
	*Enterobacter aerogenes*	2 (3.8)	3 (25.0)	0.644
	*Proteus mirabilis*	1 (1.9)	0 (0)	
	*Serratia marscecens*	2 (3.8)	0 (0)	
	*Acinetobacter baumannii*	2 (3.8)	0 (0)	
	*Stenotrophomonas maltophilia*	1 (1.9)	0 (0)	
	*Citrobacter freundii*	0 (0)	1 (8.3)	
SIBO with IBS (*n* = 93)	*Escherichia coli*	22 (31.4)	6 (26.1)	
	*Enterobacter cloacae*	9 (12.9)	0 (0)	
	*Klebsiella pneumoniae*	8 (11.4)	6 (26.1)	
	*Enterococcus faecium*	4 (5.7)	0 (0)	
	*Staphylococcus aureus*	3 (4.3)	1 (4.3)	0.125
	*Enterococcus faecalis*	3 (4.3)	2 (8.7)	
	*Enterobacter aerogenes*	3 (4.3)	2 (8.7)	
	*Klebsiella oxytoca*	3 (4.3)	1 (4.3)	
	*Pseudomonas aeruginosa*	3 (4.3)	0 (0)	
	*Acinetobacter baumannii*	2 (2.9)	0 (0)	
	*Serratia marscecens*	1 (1.4)	2 (8.7)	

*P* values indicate differences in the distribution of bacterial species between patients wihout and with history of PPI intake

**Table 5:** Linkage between IBS, SIBO and type of PPI intake

		SIBO (-) (n, %)	SIBO (+) (n, %)	*p*	OR (95 % CIs)
None	IBS (-)	478 (80.9)	53 (43.4)	<0.0001	5.50 (3.64–8.32)
	IBS (+)	113 (19.1)	69 (56.6)		
Omeprazole	IBS (-)	38 (82.6)	1 (12.5)	<0.0001	33.25 (3.57–309.2)
	IBS (+)	8 (17.4)	7 (87.5)		
Esomeprazole	IBS (-)	37 (56.1)	11 (40.7)	0.043	1.82 (1.02–4.60)
	IBS (+)	29 (43.9)	16 (59.3)		
Pantoprazole	IBS (-)	9 (81.8)	0 (0)	^a^	^a^
	IBS (+)	2 (18.2)	1 (100)		

Abbreviations: *CI* confidence interval, *OR* odds ratio

^a^cannot be calculated because one value is zero

## Discussion

Current findings challenge the concept that intake of PPIs is favoring the overgrowth of bacteria in the proximal small intestine leading to symptoms compatible with SIBO like bloating and diarrhea. The rate of PPI intake was similar between patients with SIBO and patients without SIBO as SIBO was determined by small intestinal aspirate culture. This was also the case with the absolute number of isolated bacteria whereas intake of PPIs was also not associated with the likelihood of SIBO. Instead, the logistic multiple regression analysis showed that the only factors independently associated with SIBO were the presence of IBS, T2DM and age ≥60 years. Gastritis and BMI ≥22 kg/m^2^ were protective from SIBO.

There is a traditional concept that alteration of gastric pH, as achieved after long-treatment with PPIs, can prime bacterial overgrowth and lead to SIBO. Despite this concept, existing evidence on the association of SIBO with PPI intake is not-clear cut. Quantitative culture results of the duodenal aspirates from 675 subjects who underwent upper GI endoscopy were retrospectively analyzed in relation with the history of PPI intake. Aspirates were cultured both for aerobe and anaerobe bacteria and patients were divided into those with negative aspirates, with intermediate aspirates yielding less than 10^5^ cfu/ml and with abnormal aspirates yielding more than 10^5^ cfu/ml. History of PPI intake was positively linked with the presence of intermediate aspirates; surprisingly it was not associated with abnormal aspirates [6] showing that many factors other than intake of PPI prime the development of SIBO. In another study of 150 participants, a prospective design was followed. Participants underwent not only quantitative culture of their duodenal aspirate for the diagnosis of SIBO but manometry as well to identify intestinal dysmotility. Dysmotility and PPI intake were independently associated with a greater frequency of SIBO. However, the investigators did not provide data of an interaction between PPI intake and dysmotility for the generation of SIBO so it cannot be disclosed whether patients under PPIs were suffering from dysmotility as well [11].

Prospective treatment of 52 patients with GERD with esomeprazole for 6 months was associated with the development of symptoms compatible with SIBO like bloating, flatulence and abdominal pain. In these same symptom-positive patients, SIBO was more frequent by the glucose breath test. Although findings suggest an association between PPI intake for 6 months and development of SIBO [12], it should be outlined that the study did not have a comparator arm. Moreover, patients with GERD were excluded from our study so that direct comparisons cannot be done.

It should be underscored that one main advantage of the presented analysis is the consideration of time of PPI treatment that is missing from all former publications. Three main limitations of the study should be reported. The first is the lack of culture for anaerobes. The second is the small subgroups of patients with SIBO and IBS despite the large number of performed endoscopies. The third is the lack of use of a method for prevention of contamination during endoscopy. However, the aerobic bacteria isolated from the duodenum were mainly species usually inhabiting the large bowel. This makes the chance for contamination unlikely.

The lack of association between intake of PPIs and SIBO reinforces the recently developed therapeutic concept for SIBO eradication as a means for management of IBS. Although our study failed to identify an association between PPI intake and SIBO, it clearly showed a positive association between SIBO and IBS particularly with the IBS-D subtype. Although factors like T2DM, age above 60 years, gastritis and BMI above 22 kg/m^2^ may modulate SIBO, the association between SIBO and IBS is independent from these factors. As a consequence, eradication of SIBO may be part of the algorithm for the management of IBS. A recent meta-analysis has confirmed treatment benefit in IBS with antibiotic treatment; this is pronounced with the non-absorbable rifaximin antibiotic [13]. Results of the big TARGET1 and TARGET2 randomized clinical trials were not included in this meta-analysis. In these two trials, oral rifaximin 550 mg tid for ten days improved considerably signs of IBS compared with placebo when given in patients with IBS without constipation (40.8 % versus 31.2 %, *p* = 0.01 in TARGET 1 trial; and 40.6 % versus 32.2 %, *p* = 0.03 in TARGET 2 trial) [14]. The in vitro activity of rifaximin was studied against the bacteria isolated from 117 of the patients included in our prospective cohort. To study the over-time killing effect of rifaximin, bile salts were added in the growth medium to simulate the intestinal environment. Pronounced time-kill effect was found at a concentration of 500 μg/ml against isolates of *Escherichia coli, Klebsiella pneumoniae* and *Enterococcus faecalis* that caused SIBO in our patients [15]. This concentration is considerably lower than the concentration of the drug found in the stool [16].

## Conclusions

Analysis of a cohort of 897patients with prospective quantitative culture of the duodenal aspirates did not find any association between SIBO and intake of PPI. Instead the association between IBS and SIBO was completely independent from PPI intake. Although gastritis was protective against SIBO, results show that PPI intake cannot prime SIBO.

## Abbreviations

BMI, body mass index; CFU, colony forming units; CI, confidence interval; GERD, gastroesophageal reflux disease; IBS, irritable bowel syndrome; IBS-C, irritable bowel syndrome predominant constipation; IBS-D, irritable bowel syndrome predominant diarrhea; IBS-M, irritable bowel syndrome mixed-type; OR, odds ratio; PPI, proton pump inhibitors; SIBO, small intestinal bacterial overgrowth; T2DM, type 2 diabetes mellitus
